# Fate of filter materials and microbial communities during vermifiltration process

**DOI:** 10.1016/j.jenvman.2019.04.076

**Published:** 2019-07-15

**Authors:** Amare T. Adugna, Harinaivo A. Andrianisa, Yacouba Konate, Amadou H. Maiga

**Affiliations:** a Addis Ababa Science and Technology University, AASTU, College of Biological and Chemical Engineering, Department of Environmental Engineering, P.O. Box 16417, Addis Ababa, Ethiopia; b International Institute for Water and Environmental Engineering, 2iE, 01 BP 594, Ouagadougou 01, Burkina Faso

**Keywords:** Cow dung, *Eudrilus eugeniae*, Filter materials, Sawdust, Vermifiltration, VF1, Sawdust Vermifilter 1, VF2, Sawdust Vermifilter 2, VF3, Cow dung Vermifilter 3, BOD_5_, Biological Oxygen Demand, tCOD, Total Chemical Oxygen Demand, dCOD, Dissolved Chemical Oxygen Demand, TSS, Total Suspended Solids, NO_3_^−^, Nitrate, NO_2_^−^, Nitrite, PO_4_^3−^, Orthophosphate, NH_4_^+^, Ammonium, MC, Moisture Content, VS/TS, Volatile Solids to Total Solids, TKN, Total Kjeldahl Nitrogen, CFU, Colony Forming Unit

## Abstract

The fate of filter materials and microbial communities during the vermifiltration process were studied for 5 months while treating the concentrated greywater. Four filters were filled with 10 cm gravel of which a layer of medium size gravel (5 cm thickness, aggregate size 20–40 mm) at the bottom and a layer of coarse gravel (5 cm thickness, aggregate size 10–20 mm) at the top, then filled with 20 cm sand (d_60_ = 0.2 mm, d_10_ = 0.118 mm). Finally, Vermifilter 1 (VF1), control unit and Vermifilter 2 (VF2), were filled with 40 cm fine sawdust (0.05–5 mm) but Vermifilter 3 (VF3), was filled with 40 cm cow dung (0.05–5 mm). Three filters were inoculated with 200 individuals of *Eudrilus eugeniae* except for the control unit which was filled with sawdust. Five sampling ports were installed on the wall of the filters at 10 cm intervals with reference to the surface of the top layer. Three of the filters were supplied with concentrated greywater and VF1 was supplied with drinking water at the hydraulic loading rate of 16 L m^−2^.d^−1^ on batch basis, i.e., four times a day at 8:00 a.m., 11:00 a.m., 2:00 p.m. and 5:00 p.m. Weekly, samples from influent and effluent, and monthly, samples of filter materials collected via sampling ports, were collected and analyzed.The removal efficiencies of biological oxygen demand (BOD_5_), total chemical oxygen demand (tCOD), and dissolved chemical oxygen demand (dCOD) of VF2 and VF3 were 5–7% higher than the control unit, but little differences were observed in terms of total suspended solids (TSS). However, the removal efficiencies of nutrients for the control unit was slightly better than VF2 and VF3. The pH and Moisture content (MC) of filter materials increased along the depth, but percentage of volatile solids to total solids (VS/TS) decreased through time due to the high number of microbial communities and earthworms dominating the top layer compared to the bottom. The performance of VF2-sawdust was slightly better than VF3-cow dung to treat concentrated greywater.

## Highlights

• Fate of filter materials and microbial communities during vermifiltration process were studied.
• Ratio of volatile solids to total solids decreased during vermifiltration process in filter materials.
• The bacteria, actinomycetes and fungi intensified where earthworms dominated.
• VF2-Sawdust performed slightly better than the VF3-Cow dung.

## 1 Introduction

In developing countries, the concentrated greywater generated from an urban slum area is not properly collected and treated. It is usually disposed of into roads and open spaces near the residence which causes rapid deterioration in the level of sanitation and quality of human life due to higher concentration of organic and inorganic contaminants, nutrients and pathogens. There is interest in developing viable small-scale wastewater treatment technologies suitable for small communities and individual households. Vermifiltration is proven to be an environmentally and economically preferred compared to other biological treatment technologies (Shao et al., 2014; Chyan et al., 2013; Arias et al., 2005; Carballeira et al., 2017; Kumar et al., 2016). Its performance is mainly affected by the different earthworm loads (Wang et al., 2013), hydraulic loading rates (Kumar et al., 2014), and filter materials used. In vermifiltration, microbes are responsible for biochemical degradation of organic matter, whereas earthworms act as regulators (Arora and Kazmi, 2015; Liu et al., 2012).

Filter materials are important to separate pollutants from wastewater and to create conducive environment for earthworms and microbial communities (Xing et al., 2011, 2010). Depending on the experimental goal, different filter materials have been studied. For instance, Arora et al. (2014) found riverbed material and mud balls were better for high pathogen removal (Wang et al., 2010), reported a converter slag–coal cinder filter played an important role in phosphorus removal, and Xing et al. (2011) reported ceramsite is better than quartz sand. Moreover, domestic organic waste (Taylor et al., 2003; Bajsa et al., 2003), gravel, sand, soil (Sinha et al., 2008), wood chips, bark, peat, straw (Li et al., 2008), garden soil, vermicompost (Samal et al., 2018), and river bed materials, wood coal and glass balls (Kumar et al., 2015) were found to be good for removal of organic matter and nutrients.

However, all filter materials will fail at some time (Kropf et al., 1977). For instance, (Luth et al., 2011) changed the sawdust every six months in vermifiltration process for treating swine wastewater. There was 12 cm filter bed shrinkage in the vermifiltration experiment conducted by Adugna et al. (2015). Ghatnekar et al. (2010) also reported that the bedding material gradually converted into humified vermicompost. In other filtration systems, Dalahmeh et al. (2011) found that filters with bark and wood chips showed high durability while mixed mulch, compost and wheat straw were less durable. Generally, filter materials have showed physical, chemical and biological changes in many researches. Physically, the sand was grinded down by earthworms which increased the surface area and helped to ‘adsorb’ organic and inorganic pollutants from the effluent (Ghatnekar et al., 2010; Sinha et al., 2008). Chemically, the pH changed due to absorbed or precipitated chemicals and (Hughes et al., 2008) reported the earthworm could survive in pH range of 6.2–9.7.Biologically, the microbial community population was increased, and selected species of bacteria were dominantly found in vermifilter compared to non-vermifilter. Li et al. (2014) reported Aeromonadaceae, Moraxellace, Enterobacteria, and Pseudomonadaceae found in the vermifilter that belong to the gamma proteobacteria.

However, there is no research conducted on filter materials change, particularly on sawdust and cow dung, during the vermifiltration process while treating concentrated greywater. Therefore, this research aims (1) to study the changes on filter materials and microbial communities during the vermifiltration process while treating the concentrated greywater, and (2) to compare the performance of sawdust and a degraded cow dung vermifilters, and a control unit for the removal of organic and nutrient pollutants.

## 2 Materials and methods

### 2.1 Experimental set up

**Fig. 1:**
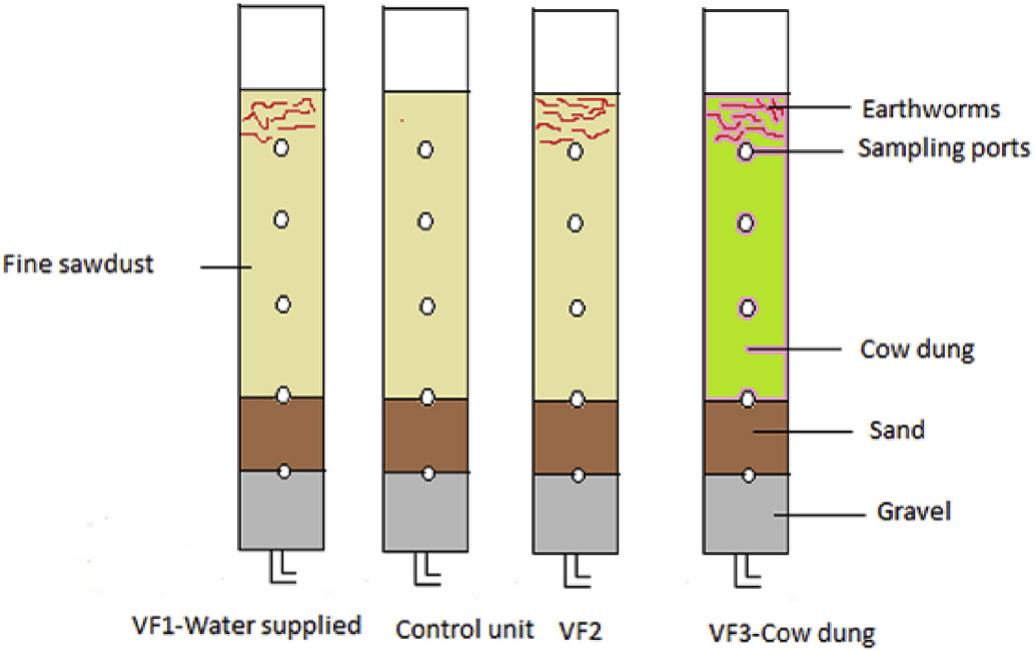
Experimental set up.

Four filters were filled with 10 cm gravel of which a layer of medium size gravel (5 cm thickness, aggregate size 20–40 mm) at the bottom and a layer of coarse gravel (5 cm thickness, aggregate size 10–20 mm) at the top, then filled with 20 cm sand (d_60_ = 0.2 mm, d_10_ = 0.118 mm). Finally, three of them, VF1, control unit, and VF2 were filled with 40 cm fine sawdust (0.05–5 mm) and the fourth filter, VF3, was filled with 40 cm cow dung (0.05–5 mm). The three vermifilters were inoculated with 200 *Eudrilus eugeniae* except for the control unit which was filled with the sawdust (Fig. 1). Five sampling ports on the wall of the filters were installed at 10 cm interval with reference to the surface of the top layer. The filters were supplied with a hydraulic loading rate of 16 L m^−2^.d^−1^ at batch basis four times a day at 8:00 a.m., 11:00 a.m., 2:00 p.m. and 5:00 p.m.

The sand had a uniformity coefficient of 1.36, an effective size of 0.118 mm and a density of 1517.6 kg/m^3^. The fine sawdust, collected from a nearby wood workshop, was composed of Khayaivorensis, Mansoniaaltissima and Miliciaexcelsa tree species. It had an average pH of 6.47, a density of 96 kg/m^3^ contained ligno-cellulosic material and rarely produced odour during its long-term biodegradation process. The filter materials washed with tap water to remove dust and other impurities. Moreover, VF1 was supplied with drinking water while others were supplied by concentrated greywater collected from a poor urban household after homogenizing as described in the procedures of Adugna et al. (2014, 2015).

### 2.2 Water quality analysis

Influent and effluent sampling, analyzing for selected Physico-chemical parameters (BOD_5,_ tCOD, dCOD and TSS) and nutrients (Ammonium (NH_4_^+^), Nitrate (NO_3_^−^), Nitrite (NO_2_^−^) and Orthophosphate (PO_4_^3−^)) were done in accordance with Standard Methods (APHA, 2005). The analysis was performed in the same day of sampling and when same day analysis was not possible, samples were stored at 4 °C for less than 24 h.

### 2.3 Earthworms and microbial community analyses

In the beginning, two hundred adult *Eudrilus eugeniae* were inoculated to three filters except for the control unit, and at the end of the experiment, earthworms were counted for change in number, weighed for mass gained, and counted for cocoons produced in each vermifilter after sorted by hand. After washing them with distilled water and dried with paper towels, the earthworms were weighed.

To identify the microbial communities working with earthworms, samples were collected from the top layer surface and five sampling ports towards the end of the experiment. Each sample was averagely 5 g, and a gram of representative sample was taken and mixed with `9 ml of sterile water using a vortex. Different dilutions were made and 1 ml of sample was spread on the autoclaved petri-dish. It was analyzed using the spread plate method with blood agar, VRBG agar, MacConkey agar, and nutritive agar media for bacteria, and Sabouraud's dextrose agar for actinomycetes and fungi. Then it was incubated for 18–24  h at 37 °C for bacteria, 10–12 days at 30 °C and 37 °C for actinomycetes, and 4–7 days at 25 °C and 28 °C for fungi (Parthasarathi et al., 2007). The different colony forming units (CFU) developed on the media were estimated and expressed as CFU x 10^4^ g^−1^ (for fungi), CFU x 10^6^ g^−1^ (for bacteria), and CFU x 10^5^ g^−1^ (actinomycetes) respectively according to the method of Baron et al. (1994). To differentiate the serological test, biochemical test, and staining (Gram's staining for actinomycetes and Lactophenol cotton blue for fungi) were used besides the incubation period and temperature. The same procedure was followed to identify them in the filter materials before the experiment was conducted.

### 2.4 Biosolids analysis

The bedding materials, fine sawdust/cow dung, with adsorbed solids from the greywater, were analyzed for VS using standard methods (APHA, 2005). The MC was determined by gravimetric method using an oven (Memmert 854. Schwabach, Germany). The pH change of filter materials was analyzed after diluting the solid sample with distilled water at 1:10 ratio and agitating using an Edmund Bühler GmbH SM-30 shaker at 200 rpm for 1 h. The C/N ratio was determined indirectly using the volatile solids (carbon) and TKN (total nitrogen), determined by the Kjeldahl method, at the beginning and end of the experiment. The porosity of the top layer was determined by volumetric method, i.e. representative samples were taken from each filter with 100 ml volumetric flask made of glass, then weighed and dried at 105 °C. After constant weight was achieved and cooled in a desiccator, a known volume of water was added until it became fully saturated, and the ratio of volume of water used to fully saturate divided by the total volume of the flask taken as the porosity. Moreover, the degradation of the sawdust components was analyzed by quantifying ash, extractives and lignin. The ash was determined using standard methods (APHA, 2005) by gravimetric methods of analysis using Carbolite Muffle Furnace, made in UK. The extractives were determined after boiling with acetone and distilled water for 6 and 2 h respectively and by drying the samples at 105 °C. The lignin was determined by mixing the extracted sample with 72% sulfuric acid that was kept in the refrigerator at 10 °C for 2 h before it was mixed with 300 ml distilled water and boiled for 1 h. After cooling, it was diluted with 150 ml of distilled water three times while being filtered. From the mass balance, it was possible to determine the holocellulose concentration.

### 2.5 Statistical analyses

Microsoft Excel 2013 was used to carry out statistical analyses and draw ﬁgures. The results were expressed as a mean ± standard deviation, and significant differences among samples were analyzed using Mann-Whitney *U* test at 5% significance level.

## 3 Results and discussion

### 3.1 Performance evaluation for the vermifilters and the control unit

The performances of vermifilters were better than the control unit for most physico-chemical parameters (BOD_5,_ tCOD, and dCOD) and NH_4_^+^. However, the control unit was slightly better for nutrient (NO_3_^−^, NO_2_^−^ and PO_4_^3-^) removal during the study period (Table 1 and Table 2). The performance of VF1 was not evaluated as it was supplied with drinking.

**Table 1:** Influent and effluent concentrations with ranges, standard deviations (SD) and average removal efficiencies for Physico-chemical parameters.

Parameters		Influent	Effluent			
			VF1	Control unit	VF2	VF3
BOD_5_(mg/L)/(%)	Average	**1234**	**16**	**78 (93.7)**^a^	**30 (97.6)**^a^	**35 (97.2)**^a^
	SD	358	8	16	12	10
	Maximum	2100	25	100	60	40
	Minimum	800	0	60	20	10
tCOD (mg/L)/(%)	Average	**2195**	**146**	**518 (76.4)**^a^	**383 (82.6)**^a^	**386 (82.4)**^a^
	SD	699	81	269	202	123
	Maximum	3520	344	906	861	549
	Minimum	1075	45	201	185	82
dCOD (mg/L)/(%)	Average	**1497**	**97**	**357 (76.2)**^a^	**254 (83)**^a^	**297 (80.2)**^a^
	SD	445	39	202	135	106
	Maximum	2180	164	632	574	474
	Minimum	570	41	142	138	69
TSS (mg/L)/(%)	Average	**1120**	**3.0**	**16 (98.6)**^a^	**7.0 (99.4)**^a^	**12 (98.9)**^a^
	SD	770	1.0	8.0	3.0	4.0
	Maximum	2960	5	38	14	16
	Minimum	352	2	5	2	4
pH	Average	**6.5**	**7.9**	**8.6**	**8.5**	**8.5**
	SD	0.5	0.2	0.2	0.2	0.2
	Maximum	7.3	8.2	8.8	8.8	8.8
	Minimum	5.8	7.6	8.3	8.2	8.2
DO (mg/L)	Average	**1.0**	**4.7**	**4.3**	**4.2**	**4.6**
	SD	0.6	1.0	1.3	0.9	1.1
	Maximum	2.2	6.8	7.7	6.2	7.1
	Minimum	0.3	3.3	2.5	2.6	3.2

a The values in bracket are the average removal efficiencies in percentage.

**Table 2:** Influent and effluent concentrations with ranges, standard deviations (SD) and removal efficiencies for nutrients.

Parameters		Influent	Effluent			
			VF1	Control unit	VF2	VF3
NH_4_^+^ (mg/L)/(%)	Average	**12**	**2**	**3(75)**^a^	**3(75)**^a^	**3 (75)**^a^
	SD	13	1.4	2.5	3.0	1.5
	Maximum	44	5	8	11	5
	Minimum	0.9	0.2	0.1	0.1	0.4
NO_3_-(mg/L)/(%)	Average	**37**	**6**	**13(64.9)**^a^	**14(83.8)**^a^	**20(83.8)**^a^
	SD	30	9	18	15	21
	Maximum	100	38	68	52	65
	Minimum	0.7	0.0	0.1	0.3	0.7
NO_2_-(mg/L)/(%)	Average	**61**	**10**	**15(75.4)**^a^	**19(68.9)**^a^	**22(63.9)**^a^
	SD	57	11	14	16	19
	Maximum	210	40	60	60	60
	Minimum	6.0	0.2	2.0	0.0	0.6
PO_4_^3−^ (mg/L)/(%)	Average	**32**	**1.0**	**17 (46.9)**^a^	**22 (31.3)**^a^	**25 (21.9)**^a^
	SD	53	2.0	29	41	42
	Maximum	199	6.0	91	141	144
	Minimum	0.6	0.0	0.1	0.01	0.3

a The values in bracket are the average removal efficiencies in percentage.

#### 3.1.1 Physico-chemical Parameters

As shown from Table 1, the average removal efficiencies for BOD_5_, tCOD, dCOD and TSS were 93.7%, 76.4%, 76.2% and 98.6% for control unit, 97.6%, 82.6%, 83.0% and 99.4% for VF2, and 97.2%, 82.4%, 80.2% and 98.9% for VF3.Generally, VF2 and VF3 had 5–7% higher removal efficiencies than the control unit except for TSS. Moreover, the effluent concentrations for physico-chemical parameters and nutrients from VF1, supplied with drinking water, showed that there was leach out of pollutants from filter media.

#### 3.1.2 Nutrients removal

The average removal efficiencies for NH_4_^+^, NO_3_^−^, NO_2_^−^ and PO_4_^3−^ are 75.0%, 64.9%, 75.4% and 46.9% for control unit, 75.0%, 62.2%, 68.9% and 31.3% for VF2, and 75.0%, 46%, 63.9% and 21.9% for VF3 respectively (Table 2). Generally, the control unit is slightly better than VF1 and VF2 in nutrient removal efficiency except for NH_4_^+^. This may be due to the occurrence of more nitrification in vermifilters that affect the better nitrate removal and change of particulate phosphorous into soluble (orthophosphate) by the activities of earthworms and microbial communities. The adsorption capacity of sawdust might also contribute for better removal in the control unit (Harmayani, 2012). found that sawdust is a very good adsorbent to remove NH_3_—N, NO_3_—N, and NO_2_—N from aqueous solution. The earthworm casts are also known for adsorption of different chemical pollutants (Prasad Kumar and Kumar, 2013). Moreover, better nitrification may be achieved due to aerobic conditions created by earthworms’ activities in the vermifilters, and the batch feeding system both in vermifilters and control unit. Similarly, Pell and Nyberg (1989) reported the complete nitrification in the top 15 cm layer of sand filter columns. However, lower performance from VF3 might be due to the less porosity and nutrient available in cow dung (Table 2). The removal of nitrate can also be due to the denitrifying bacteria in the earthworm gut (Svensson et al., 1986; Elliott et al., 1991; Matthies et al., 1999).

Though the control unit performance is slightly better, VF2 and VF3 removed 31% and 22% of the orthophosphate respectively (Table 2) which is less than Luth et al. (2011) finding, around 40%.Taylor et al. (2003) also reported the phosphorus removal by vermifilter. The better removal in VF2 may be due to the sorption capacity of filter materials, and Jiang et al. (2016) reported phosphorous removal depends on chemical reaction like ligand exchange reaction, complexation and precipitation.

Moreover, there was a significant difference (p < 0.05) for BOD_5_, tCOD, dCOD and TSS removal efficiencies between control unit and VF2, for BOD_5_ and PO_4_^3−^ removal efficiencies between the control unit and VF3, and for PO_4_^3−^ removal efficiency between VF2 and VF3 (Table 3). There were also significant differences in pH between the control unit and VF2, BOD_5_ and NO_2_^−^ concentrations between the control unit and VF3, TSS and DO concentrations between VF2 and VF3 (Table 3). To summarize, VF2 performed better than VF3 and the control unit.

**Table 3:** P-values for removal efficiencies and effluent concentrations among control unit, VF2 and VF3.

Constituent	Removal efficiencies			Concentrations		
	Control X VF2	Control X VF3	VF2 X VF3	Control X VF2	Control X VF3	VF2 X VF3
BOD_5_	2.99E-06(*)	5.1E-06(*)	0.10497	1.96E-07(*)	3.2E-07(*)	0.32119
CODt	0.015698(*)	0.220867	0.76653	0.0078(*)	0.18513	0.97467
CODd	0.045828(*)	0.832098	0.23448	0.0271(*)	0.39698	0.29765
TSS	0.000132(*)	0.057708	0.20651	0.0003(*)	0.07290	0.016(*)
NH_4_^+^	0.090558	0.069806	0.06981	0.72269	0.78283	0.65469
NO_3_^−^	0.172944	0.139313	0.32004	0.34987	0.28028	0.32552
NO_2_^−^	0.130939	0.162056	0.79331	0.06231	0.038(*)	0.30327
PO_4_^3-^	0.838387	0.00055(*)	0.003(*)	0.71536	0.09048	0.11846
DO				0.40919	0.29923	0.016(*)
pH				0.0042(*)	0.25592	0.89157

(*) p-values ≤ 0.05: sample medians are significantly different.

#### 3.1.3 Earthworm evolution

There were mature and immature earthworms and cocoons in vermifilters except in VF1 supplied with drinking water. As shown in Table 4, VF2 had 202, 75 and 83, and VF3 had 148, 35 and 20 of Adults (mature), immature and cocoons respectively. However, the number of earthworms (adults, juveniles, and cocoons) in VF1, supplied with drinking water, was zero at the end of the experiment. From total death of earthworms in VF1, the greywater was source of energy and nutrient for earthworms in addition to the VS of sawdust/cow dung in VF2 and VF3. Similarly, Ghunmi et al. (2011), Leal et al. (2007, 2011) and Zeeman et al. (2008) reported that greywater contained an easily available carbon and energy source. The number of earthworms and cocoons decreased significantly due to high temperature (24 °C–42 °C) from March to May. However, the existence of juveniles and cocoons showed that earthworms are reproducing (Xing et al., 2010) and they are bioindicators for ecological condition (Edwards and Bohlen, 1996; Kruum, 2005).

**Table 4:** Earthworm evolution in the vermifilters.

		Initial	After 5 months		
			Mature	Immature	Cocoons
VF1 Sawdust	Number	200	0	0	0
	Total wt. (gram)	110.2	–	–	–
VF2 Sawdust	Number	200	202	75	83
	Total wt. (gram)	109.1	118.2	9.3	–
VF3 Cow dung	Number	200	148	35	20
	Total wt. (gram)	114.6	89.7	4.7	–

### 3.2 Effect on filter materials

#### 3.2.1 Initial concentrations

The initial concentrations of BOD_5,_ NH_4_^+^, NO_3_^−^, PO_4_^3−^, COD, and pH for filter materials analyzed and presented in Table 5. The filter materials had already some pollutants but frequent washing with drinking water helped to remove before starting the experiment.

**Table 5:** The concentrations of some parameters in filter materials.

Filter materials	Concentrations of parameters					
	pH	NH_4_^+^ (mg/L)	PO_4_^3−^(mg/L)	NO_3_^−^(mg/L)	COD (mg/L)	BOD_5_ (mg/L)
Sawdust	6.5	2.0	0.33	0.8	529	234
Sand	6.5	0.2	0.66	0.1	0	0
Cow dung	7.9	0.4	2.65	1.3	476	150

#### 3.2.2 Average pH trend along the depth

**Fig. 2:**
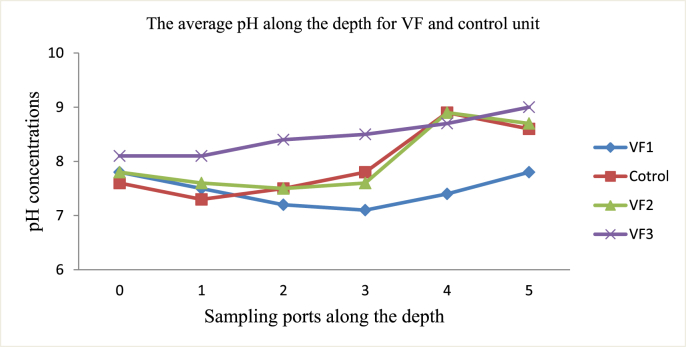
The average pH of filter materials along the depth for vermifilters and control unit.

The average initial pH values of filter materials were 6.5 for fine sawdust, 6.5 for sand, and 7.9 for cow dung. The values of pH slowly decreased for VF1 and VF2 until 30 cm depth but VF3 showed a continuous increase. However, the control unit showed both decreasing and increasing trend for the same depth (Fig. 2). The pH change in filter materials might be due to the supplied greywater, activities of earthworms and microbial communities. On the surface of sand layer, the pH increased significantly which might be due to the accumulation, precipitation, and transformation of bicarbonate, carbonate and hydroxide.

#### 3.2.3 Porosity of the bedding material

**Fig. 3:**
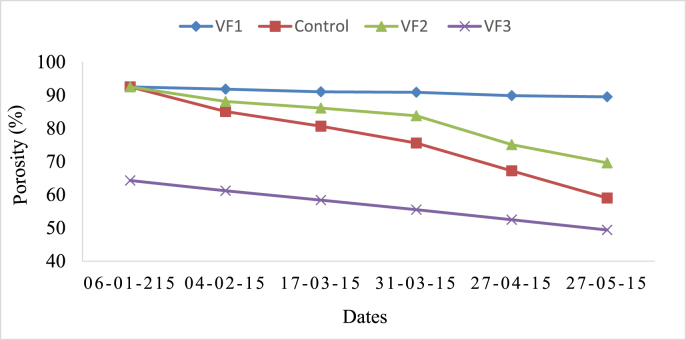
The top layer porosity change in all filters through time.

Porosity decreased in all filters, but at a slower rate for vermifilters than the control unit (Fig. 3). The decrease in porosity might be due to accumulation of slowly degraded organic and inorganic solids from greywater, cast accumulation, and biomat formation. The digestion of accumulated solids and fine sawdust by earthworms reduced the filter materials size which might slowly reduce the porosity.

#### 3.2.4 Volatile solids and moisture content change

**Fig. 4:**
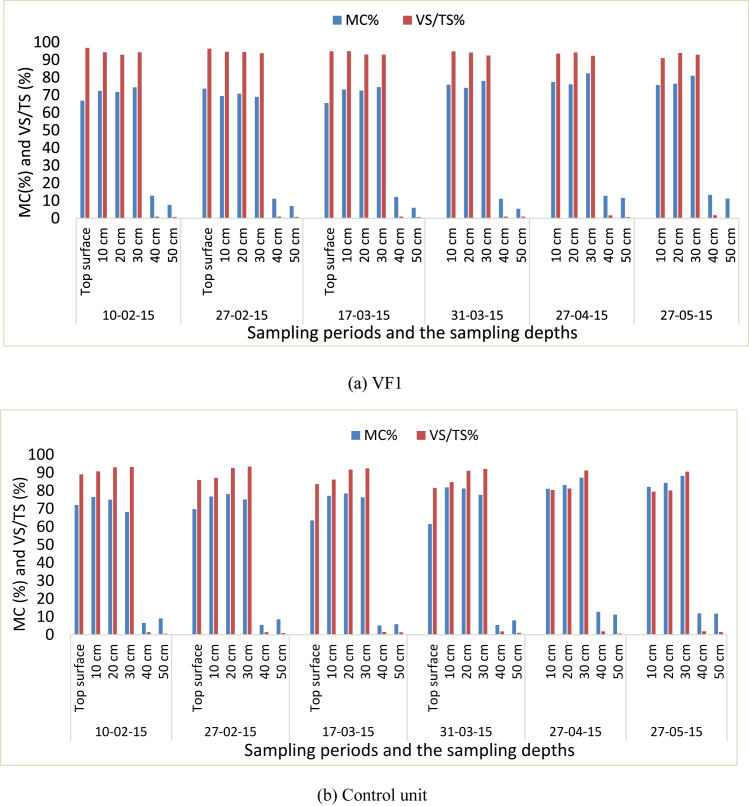

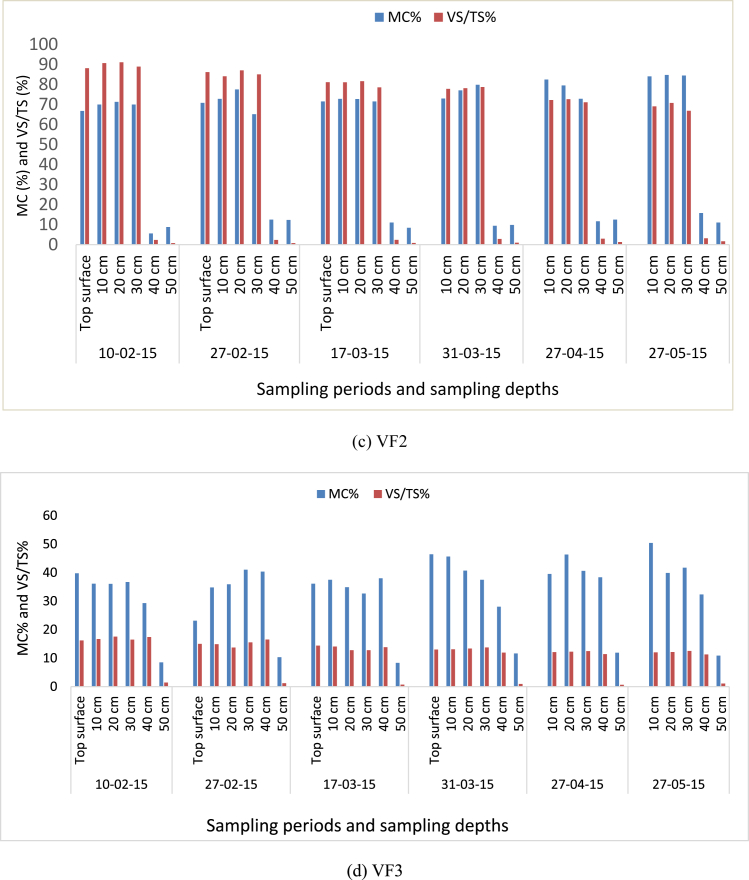
The volatile solids and moisture content along the depth of VF1 (a), Control unit (b), VF2 (c) and VF3 (d) for different dates.

Fig. 4 presents the VS/TS (%) and MC (%) of the filter materials taken from the surface of top layer and five sampling ports of each filter. The VF1 showed little decrease in VS/TS and some increment of MC throughout the research period. However, for the rest of the filters, there was more decrease in VS/TS on top layer and decreased at decreasing rate along depth through time as more VS (carbon) consumed where earthworms and microbes dominate. Generally, VS/TS (%) decreased through time for each sampling points.

The optimum moisture content was in the range of (60%–70%) during the study time, and Sinha et al. (2010) reported similar findings (Fig. 4). The bedding materials and additional solids from greywater were grinded into small particles (<2 μm) by the earthworm gizzard which enhanced the surface area for microbial action (Singh and Sharama, 2002; Aira et al., 2007; Suthar and Singh, 2008).

#### 3.2.5 C/N ratio and fine sawdust component degradation

**Fig. 5:**
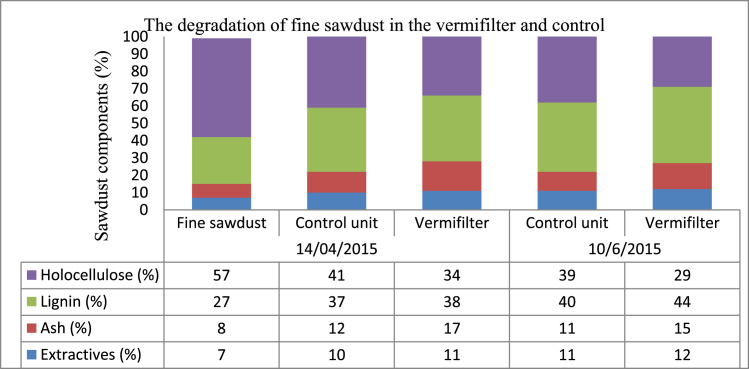
The degradation of fine sawdust in the vermifilter and control unit.

There was a significant reduction of cellulose from the bedding material (fine sawdust) in the vermifilters than the control unit (Fig. 5). The cellulose degradation in the control unit showed that bacteria are responsible for the degradation and Morgan and Burrows (1982) reported similar finding.

The C/N ratio of the bedding material (fine sawdust) changed by the activities of earthworms and microbial communities in the vermifilter, and only by microbial communities in the control unit. As a result, the C/N ratio changed from 247 to 70, 84 to 14, 79 to 25 and 17 to 11 for VF1, Control unit, VF2 and VF3 respectively (Table 6). Therefore, the lower C/N ratio in the control unit might be due to the adsorbed and less utilized nitrogen components compared to VF2 as the earthworms utilized additional nitrogen components besides the less degradation of VS in the control unit. However, the cow dung had more nitrogen components from beginning which might decreased the C/N ratio further. Samples collected from the top layer within 10 cm depth. Similarly, Domineguez and Edwards (2004) reported the earthworms reduced the size of the bedding material, gradually reducing of C/N ratio and increased surface area exposed to the microorganisms that facilitated the degradation.

**Table 6:** C/N ratio, TKN and VS at the initial and end of experiment.

		The vermifilters and control unit			
		VF1	Control	VF2	VF3
VS (mg/kg)	Initial (20/01/2015)	968	891	881	162
	Final (02/06/2015)	935	795	691	153
TKN (mg/L)	Initial (20/01/2015)	4	11	11	10
	Final (02/06/2015)	13	55	27	13
C/N ratio	Initial (20/01/2015)	247	84	79	17
	Final (02/06/2015)	70	14	25	11

#### 3.2.6 Microbial communities identification and enumeration

In each filter, the number of identified bacteria, actinomycetes, and fungi are presented in Table 7 below. In the vermifilters, there was greater bacterial population variation along the depth compared to the control unit. Arora et al. (2014) reported the same trend for bacterial population change in the vermifilter but not for the control unit. Besides, VF2 had 5 times more population of bacteria than the control unit that might be due to the activities of earthworms. The dominantly available bacteria phylum in vermicomposting and vermifiltration is *Proteobacteria* (Danon et al., 2008; Fracchia et al., 2006; Vivas et al., 2009; Zhao et al., 2010). There were also more fungi in the vermifilters due to the aeration created by earthworms. Parthasarathi et al. (2007) found that the diversity of fungi, bacteria, yeast, actinomycetes and protozoa in the gut and casts of *Eudrilus eugeniae*.

**Table 7:** Microbial communities enumeration along the depth.

(a) At the end of the experiment												
Sampling ports	Bacterial community (10^11^ CFU/g^−1^), Actinomycetes (10^7^ CFU/g^−1^) and Fungi (10^6^ CFU/g^−1^)											
	VF1			Control unit			VF2			VF3		
	Bacteria	Actimacytes	Fungi	Bacteria	Actimacytes	Fungi	Bacteria	Actimacytes	Fungi	Bacteria	Actimacytes	Fungi
1	4.6	8.8	31.3	45.2	14.5	38.3	–	–	–	27.0	15.0	15.0
2	3.5	11.3	12.5	42.8	18.8	34.4	205.0	27.8	37.2	38.3	23.8	23.8
3	3.4	10.0	11.3	27.1	16.4	33.6	154.2	19.7	34.2	36.6	31.3	31.3
4	1.1	6.0	6.0	13.0	5.0	5.0	42.6	6.7	32.6	23.7	12.9	12.9
5	1.6	3.5	4.1	10.4	6.7	6.7	5.3	5.7	5.3	5.7	6.9	7.7

(b) Microbial communities in the filter materials			
Bacterial community (10^6^ CFU/g^−1^), Actinomycetes (10^5^ CFU/g^−1^) and Fungi (10^4^ CFU/g^−1^)			
	Bacteria	Actimacytes	Fungi
Sawdust	5100	80	120
Sand	1200	100	140
Cow dung	5300	245	10

## 4 Conclusions

The filter materials change during vermifiltration process may be due to the activities of earthworms and microbial communities, concentrated greywater, and nature of filter materials. The porosity decreased in all filters but at slower rate for vermifilters. The VS/TS ration reduced more on top layer where earthworms and microbes dominate, and decreased at decreasing rate along depth.VF2-sawdust showed maximum reduction of VS, 881 to 691 mg/kg while VF3-cow dung showed minimum reduction, 162 to 153 mg/kg. The optimum moisture was also in the range of (60%–70%) for the majority of the time which is similar to (Sinha et al., 2010) finding. The pH values were slowly decreased until 30 cm depth for VF1 and VF2, but VF3 showed a continuous increase, and the control unit showed both decreasing and increasing trend for the same depth. The cellulose reduction was significant in vermifilters than the control unit, and C/N ratio was changed from 247 to 70, 84 to 14, 79 to 25 and 17 to 11 for VF1, Control unit, VF2 and VF3 respectively. Moreover, the most common microbial communities working with earthworms, i.e. fungi, bacteria, and actinomycetes were identified in the sample of filter materials at different depths. More bacteria were observed in VF2 and VF3 compared to others which may be due to the activities of earthworms. The bacterial distribution in vermifilters and control unit was higher at the top and decreased to the bottom, but at higher rate for the vermifilters. Then, changes in sawdust are more than the cow dung for most parameters.

Finally, the vermifilters performed better for BOD_5_, tCOD, dCOD, TSS and NH_4_^+^ removal, and the control unit was slightly better for NO_3_^−^, NO_2_^−^ and PO_4_^3−^ removal. Generally, VF2 and VF3 had 5–7% higher removal efficiencies than the control unit, except for TSS. Additionally, VF2 performed slightly better thanVF3 which might be due to the sawdust adsorption capacity, and availability of more pollutants and lower porosity in the cow dung. Therefore, this research recommended VF2-sawdust compared to VF3-cowdung in treating concentrated greywater.
